# A study of degradable orthopedic implant: An insight in magnesium metal matrix composites

**DOI:** 10.1016/j.heliyon.2022.e10503

**Published:** 2022-09-01

**Authors:** Adedotun Adetunla, Anthony Fide-Akwuobi, Henry Benjamin, Adebayo Adeyinka, Adenike Kolawole

**Affiliations:** a Department of Mechanical and Mechatronics Engineering, Afe Babalola University, Ado, Nigeria; b Department of Mechanical Engineering, Ekiti State University, Ado- Ekiti, Nigeria; c Department of Mechanical Engineering, Ajayi Crowther University, Oyo, Nigeria

**Keywords:** Magnesium alloy, Mechanical properties, Orthopedic implants, Reinforcement

## Abstract

Majority of the properties required for orthopedic implants operation are demonstrated by magnesium and its alloys, however the metal degrades rapidly in the body's environment. Therefore, a magnesium-based metal matrix composite capable of safely and gradually degrading in the body within the required healing time is required, thereby eliminating the need for a secondary surgery. In this study, three AZ31 Mg alloy samples with 50% reinforcement of Calcium Carbonate Powder, 25% reinforcement of CaCo_3_, and no reinforcement (As-received) were developed via stir-casting technique. X-ray Fluorescence was used to determine the chemical composition of the alloy while the microstructural characterization was determined by SEM. Furthermore, tensile, impact, corrosion and hardness tests were performed to determine the mechanical properties of the composites. The findings show that the newly fabricated alloy (AZ31B Mg/CaCO_3_) has a good chance of being employed in orthopedic applications where corrosion resistance is critical, as it shows an improved tensile and hardness properties when compared with the unreinforced Az31 Mg alloy.

## 1 Introduction

Bone healing is a natural process; therefore, re-alignment is used to correct fractures and displacements caused by physical trauma, osteoporosis, or any other underlying ailments. Adults may experience elongated bone healing periods compared to that of the lower age groups because they contain less periosteum, which is a thick layer of interconnected tissues abundant in children.

Selective metals have been incorporated by orthopedics to give structural support to the damaged parts in form of screws or grafts which must be implanted through a surgical process and removed after healing through a second surgical process [1, 2, 3, 4].

An ideal material selection for this process should satisfy the mechanical, biocompatibility, low degradation, corrosion, and good wear resistance properties. Despite these considerations, significant mechanical strength to withstand biomechanical loads is expected of these implants [5, 6]. Most of the properties required for orthopedic implants operation are demonstrated by magnesium and its alloys. Haghshenas 2017 [7], states that the metal possesses an elastic modulus of about 41–45 GPa, like that of the cortical bone. It also has a good strength to weight ratio required for rigidity.

However, the metal by itself degrades quickly in the body's environment even before the required healing time. Magnesium in its pure state has some drawbacks. According to [8], the rate of decomposition of pure magnesium in vivo is particularly high, limiting its use in implant applications that are exposed to bodily fluid. This (i) compromises the mechanical integrity of the implant before the broken bone tissue heals completely, and (ii) releases a considerable amount of hydrogen gases, causing subcutaneous bubbles and delaying the healing process of the damaged region. Due to the difficulties of employing pure magnesium in bio-applications, magnesium alloys and magnesium-based composites for biodegradable implants have been created. Biodegradability is a property of materials such that they can disintegrate and diffuse gradually in vivo without any toxic effects [9, 10, 11].

Consider an individual undergoing therapy due to a bone fracture, a surgery is needed to insert the bone implants which remain throughout the healing span, while another surgery may be required to remove the implants after healing, which is an additional cost and the general trauma associated with surgeries. The aim of the study is to develop a Magnesium based metal matrix composite capable of safely and gradually degrading in the body within the required healing time thereby eliminating the need for a secondary surgery. The results obtained from this study are discussed extensively.

## 2 Materials and methodology

A design of the experimental approach was carried out, to explore the effect of Calcium Carbonate (CaCO_3_) powder on the grade AZ31B Magnesium alloy for applications such as orthopedic implants.

**Figure 1:**
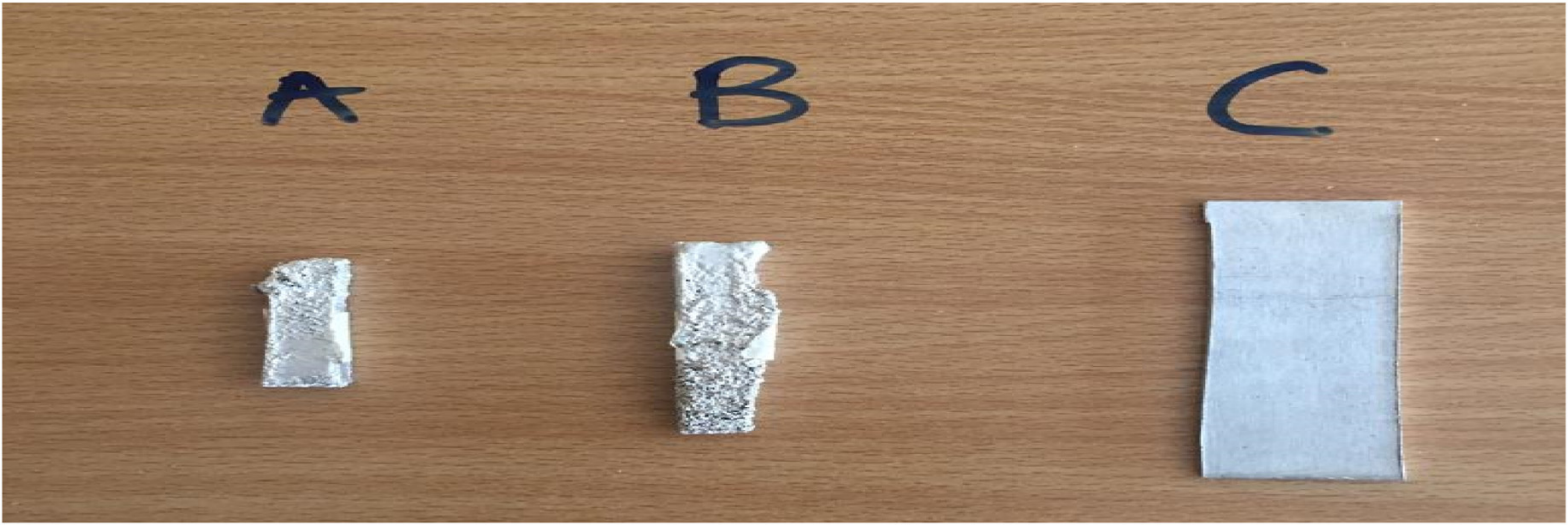
Stir-cast of AZ31BMg–CaCO3 samples.

The base metal employed in this study is the AZ31 Magnesium (Mg) alloy with Calcium Carbonate powder (CaCO_3_) used as its reinforcement. The composition of the CaCO_3_ powder in the casting process were varied according to weight, resulting in three cast samples A, B and C as seen in Figure 1.

Sample A contained 50% of the base metal and the reinforcement each, while Sample B contained 75% of the base metal and 25% of the reinforcement, while C, used as the control sample, contained 0% of the reinforcement. The resulting samples were surface smoothened after casting. Table 1 shows the varied compositions of the cast AZ31BMg–CaCO_3_ composites.

**Table 1:** Varied compositions of the cast AZ31BMg and CaCO3 composite.

Sample	AZ31BMg		CaCO_3_	
	(%)	(g)	(%)	(g)
A	50	16.35	50	16.35
B	75	17.95	25	13.46g
C	100	15.7	-	-

**Figure 2:**
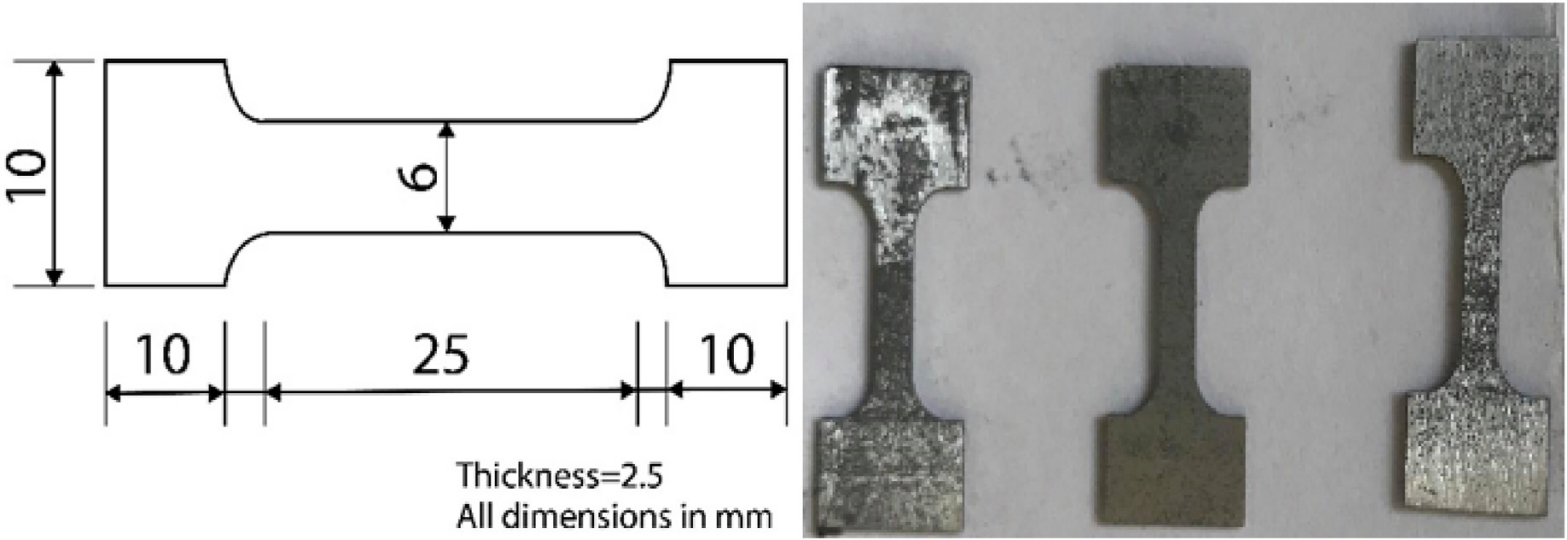
The dimension of the Tensile Samples.

X-Ray Fluorescence (XRF), which is based on surface investigation, was used to assess the elemental composition of materials. The as-received CaCO_3_ powder was characterized using a Particle Size Analyzer and the average particle size was recorded as 50μm. It is a precipitated powder formed from the breakdown of limestone to calcium oxide followed by a recarbonization process. The tensile test were conducted in compliance with ASTM standard as shown in Figure 2.

The tests were performed at 25 °C (room temperature) using an electronic tensile machine with 2.0 × 10^−3^ s^−1^ strain rate. For the hardness test, the cast samples were flattened and polished for the hardness test which was performed using the Brinell Hardness Tester with a tungsten carbide ball indenter of 1.57mm diameter according to the ASTM E384 standard. A load of 100 kgf was used with a dwell time of 15 s. The readings were then taken from the scale while following the necessary precautions. The indentation sizes were examined using a Brinell microscope after the samples A, B, and C had been indented respectively. The expression for calculation of Brinell Hardness Number (BHN) is given in Eq. (1).

(1) $$BHN=\frac{P}{\frac{\pi D}{2}\left(D-\sqrt{D^{2}-d^{2}}\right)}$$

Where;

‘D’ is the ball indenter diameter.

‘d’ is the average indentation diameter.

‘P’ is the applied load.

**Figure 3:**
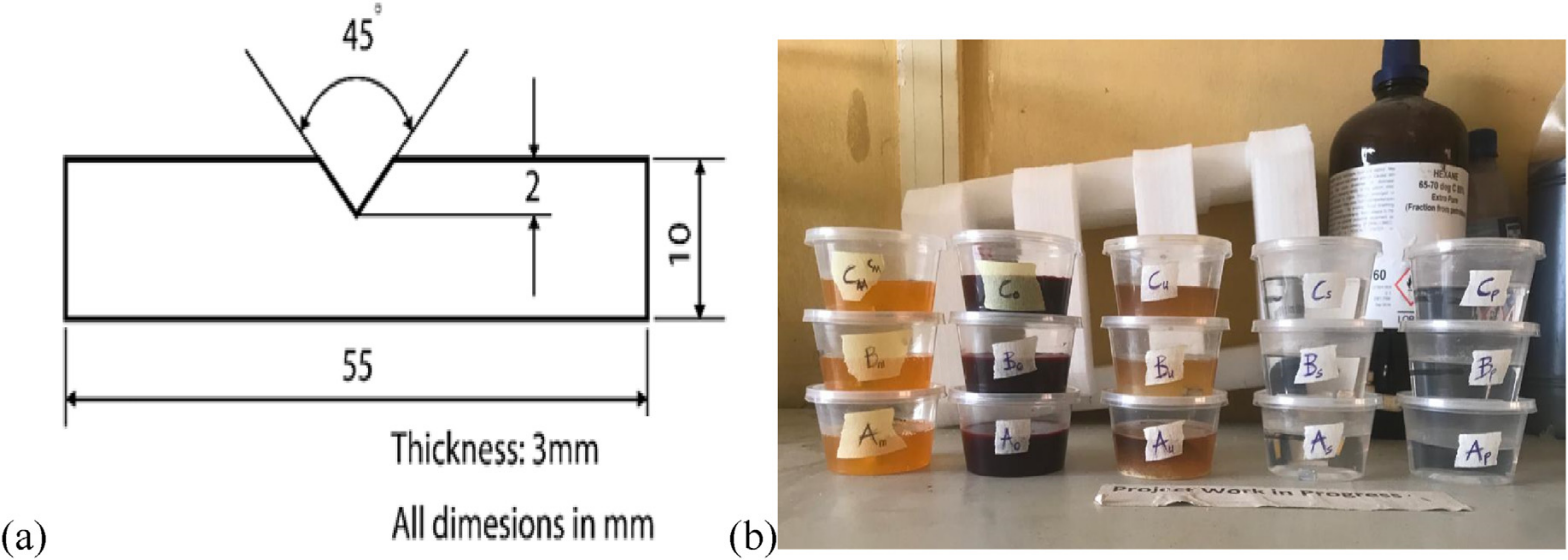
(a) Dimension of sample for impact test, (b) Samples in various corrosive media.

The aggregation of the Calcium Carbonate (CaCO_3_) reinforcement particles within the metal matrix, as well as their grain sizes, were observed by SEM. The Phenom ProX SEM model was used to investigate particle dispersion and surface morphology at magnifications of 100μm, 80μm, 50μm, and 20μm. The samples were adhered on a stub, fixed with double adhesives, then coated with an ultra-thin 5mm layer of gold via a Quorum technologies model Q150R sputter coater. It was then moved to the chamber of the SEM machine, where it was viewed using navigational camera (Navcam) for focusing and fine adjustments. To determine the performance of the magnesium metal matrix composite under shock loading, the samples were machined to the dimensions in Figure 3a. V-Notches of depth 2mm were engrooved into the samples using a triangular file. The test was performed using a Charpy Impact Tester at room temperature observing all necessary precautions.

Weight reduction method was adopted to determine the corrosion resistance of the composite samples in pure water (p), sweat (s), urine (u), blood (o), and plasma (m). Fifteen samples overall were cut from specimen A (50% reinforcement), B (25% reinforcement), and C (0% reinforcement) respectively as shown in Figure 3b. 50ml of each corrosive media were poured into fifteen plastic containers appropriately labeled with the sample composition as shown in Table 2. The masses of the samples were measured for weight loss over a period of 48 days with intervals of 5 days each. Samples such as plasma, urine and blood were taken from one of the authors of this paper, who consented to take part in this experiment. The ethical clearance committee for research and development at the Ekiti State University, Ado, Nigeria issued an ethical clearance certificate to this effect in approval of this experiment.

**Table 2:** Preparation of corrosion test in various corrosive media.

Medium	Sample	Mass of Container (G)	Volume of Medium (Ml)	Mass of Container + Medium (G)	Mass of Container + Medium + Sample (G)
WATER	Ap	9.77	50	71.98	72.6
	Bp	9.73	50	84.81	86.05
	Cp	9.72	50	81.17	81.75
SWEAT	As	9.73	50	91.44	91.98
	Bs	9.59	50	96.19	97.49
	Cs	9.71	50	96.94	97.42
URINE	Au	9.72	50	80.88	81.53
	Bu	9.64	50	80.49	81.95
	Cu	9.65	50	71.31	71.93
BLOOD	Ao	9.72	50	80.13	80.73
	Bo	9.74	50	78.6	79.78
	Co	9.5	50	87.73	88.37
PLASMA	Am	9.76	50	71.83	72.46
	Bm	9.88	50	73.26	74.07
	Cm	9.65	50	69.96	70.51

## 3 Results and discussion

The results and discussion of the analyses performed following the Stir Casting process, utilizing various process parameters and instruments, are presented, and discussed in this section.

### 3.1 Microstructural analysis

The compound composition of the base metal determined using the energy-dispersive XRF (ED-XRF) method with base metal comprising 96 percent Magnesium Oxide, whereas additional elements tagged as alloying elements make up less than 4% of the alloy. A particle size analyser was used to characterize the as-received CaCO_3_ powder with purity greater than 99% and the average particles size was found to be 50μm which is in agreement with similar studies [12, 13, 14].

**Figure 4:**
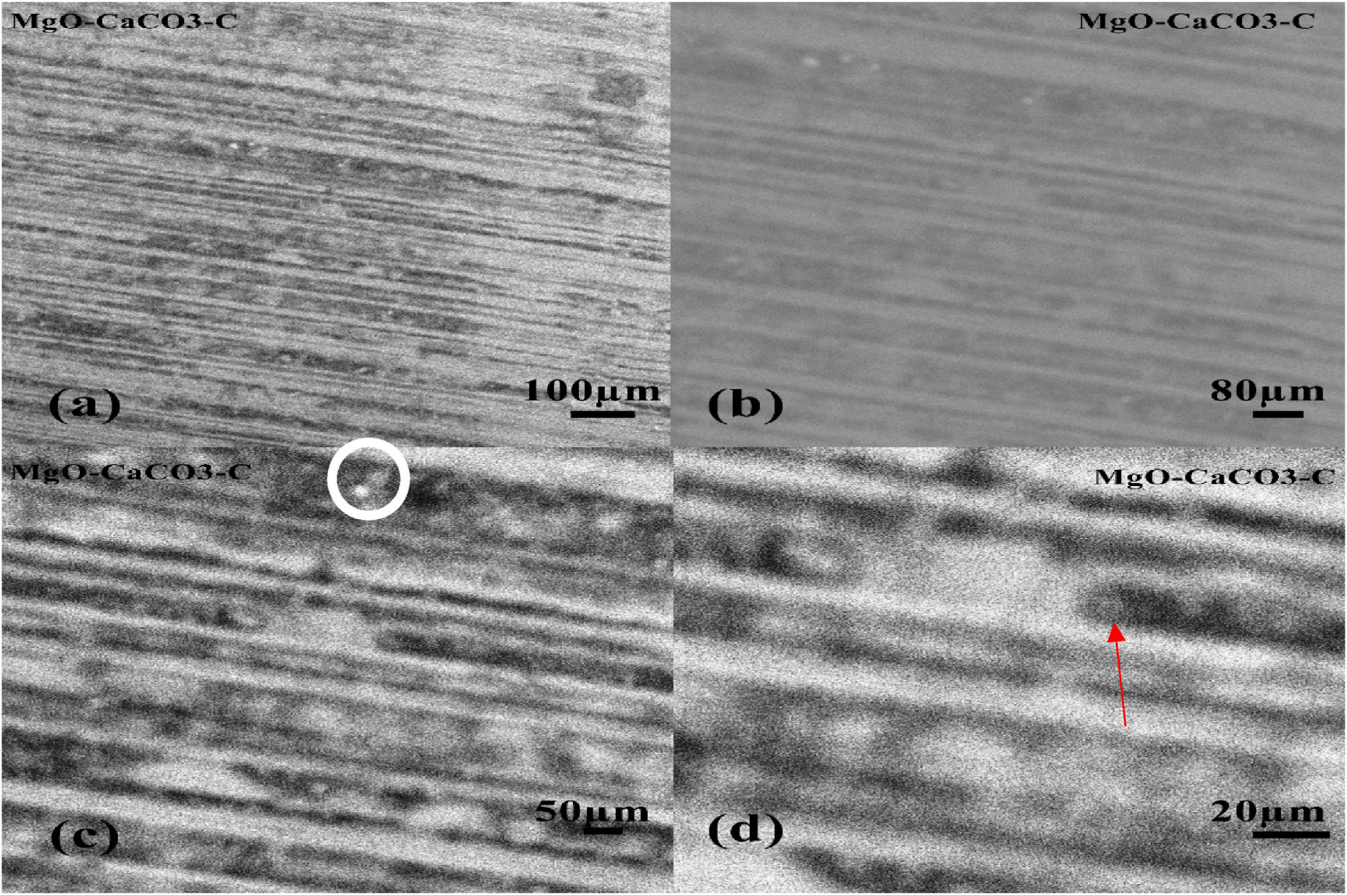
SEM micrograph of sample C (control sample) (a) 100 μm, (b) 80 μm, (c) 50 μm, (d) 20 μm.

The surface of the parent material, AZ31B Magnesium alloy, was analysed using the Phenom ProX SEM machine with magnifications of 100μm, 80μm, 50μm, and 20μm as shown in Figures 4a, 4b, 4c, and 4d respectively. The darker precipitates in the as-received alloy have a fine continuous distribution across the grains and inside grain borders, with grain boundaries that are elongated or stretched, indicating that the cell configuration of the precipitates is eutectic. The outlined white spot in Figure 4c indicates the presence of undissolved elements or impurities in the alloy. However, it is negligible as its occurrence is rare throughout the microstructure.

**Figure 5:**
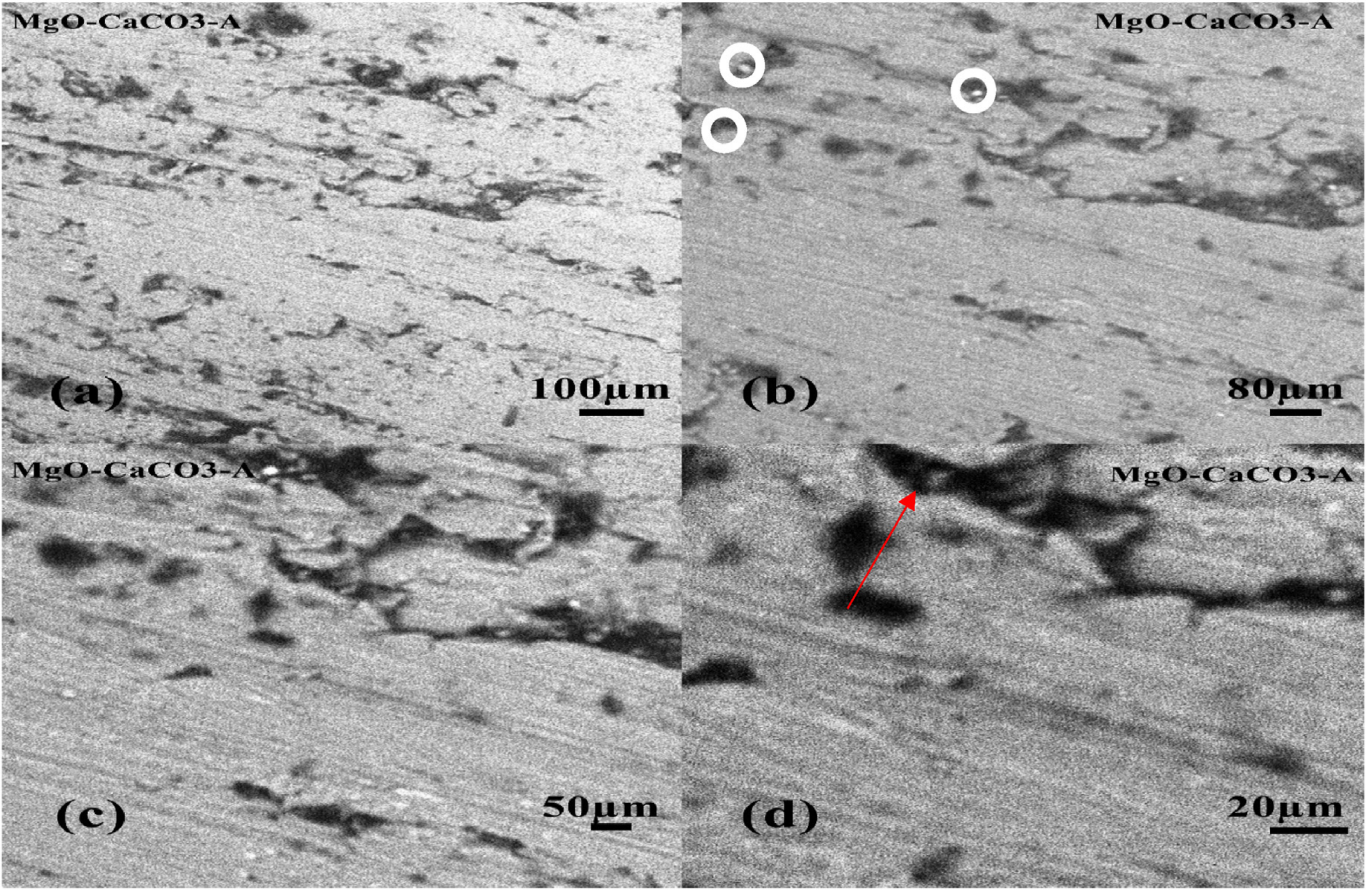
SEM micrograph of sample A (50% reinforced) (a) 100 μm, (b) 80 μm (c) 50 μm, (d) 20 μm.

Figures 5a, 5b, 5c and 5d shows the microstructural imaging of Sample A (50% reinforced) at various magnifications. The outlined white spot in Figure 5b indicates the presence of the undissolved CaCO_3_ reinforcement in the matrix due to differences in their melting point. As seen in the parent material, its appearance is also negligible, therefore a fine distribution of the reinforcement throughout the matrix was obtained.

**Figure 6:**
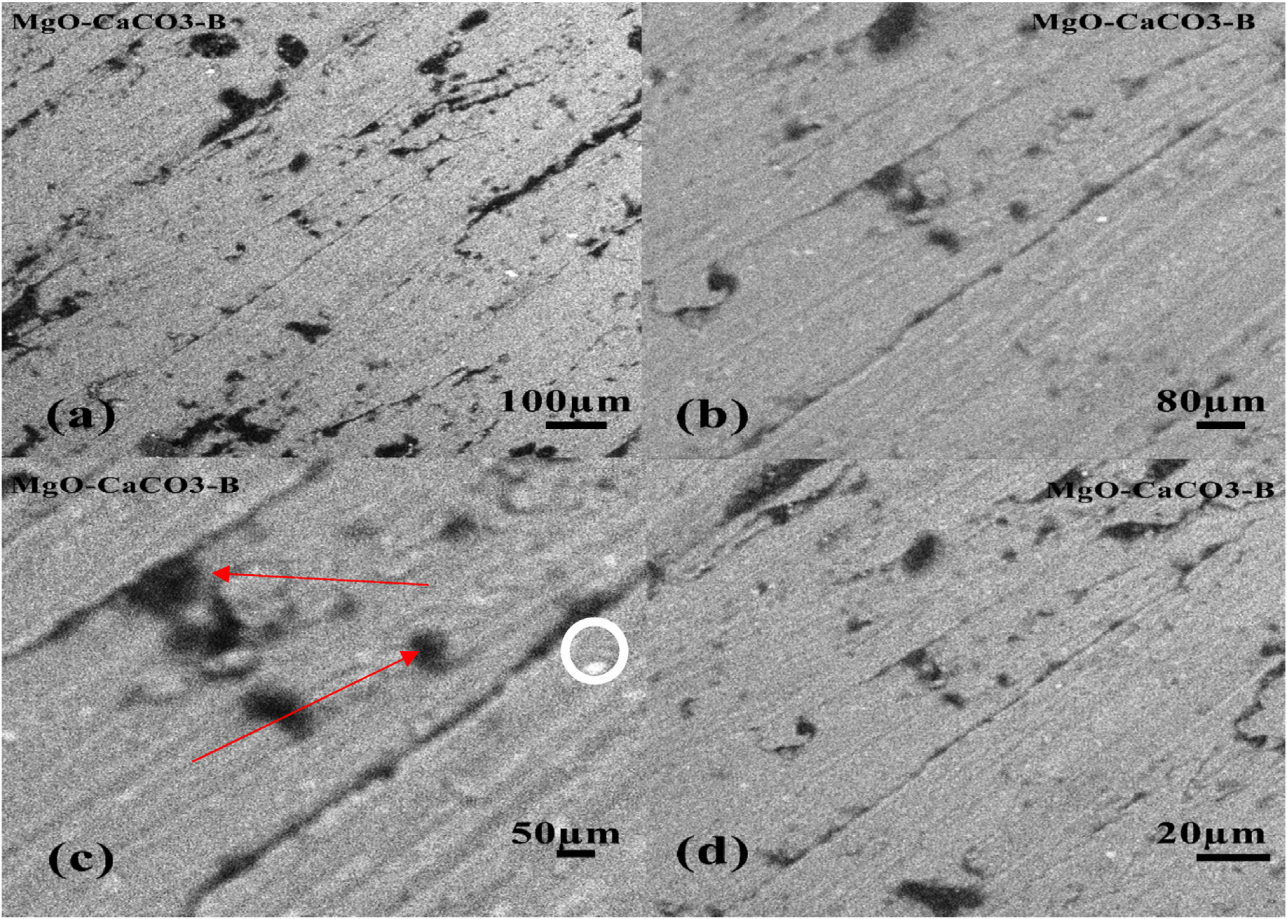
SEM micrograph of sample B (25% reinforced) (a) 100 μm, (b) 80 μm, (c) 50 μm, (d) 20 μm.

The microstructural imaging of Sample B (25% reinforced) at various magnifications are represented in Figures 6a, 6b, 6c and 6d. There is an occurrence of microstructural defect like tunnel holes as observed in Sample A. However, less undissolved particles and a finer distribution than Figure 6d can be observed from the image. Due to the dynamic recrystallization which occurs at the stir zone, it has been established that grain refinement during the process can lead to enhanced mechanical properties, such as the hardness value, using the Hall-Petch equation [15]. The SEM results in this study shows that the reinforcing particles were evenly distributed and no much microstructural defects, the few aglomeration of particles observed in this study could be as a result of difference in the melting point of both alloys, which is associated with casting method as observed by some previous works [16, 17, 18].

### 3.2 Tensile test results

**Figure 7:**
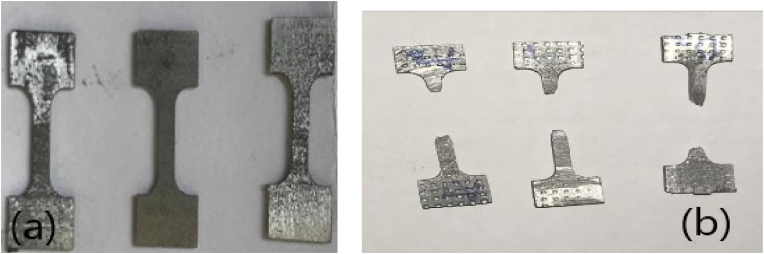
Tensile specimen (a) before fracture (b) after fracture.

The impact of process variables on the tensile characteristics of the casted composite samples was investigated. Figures 7a and 7b shows the images of the machined specimens before and after testing, with all samples fractured at different loads. Table 3 shows that all reinforced samples had better tensile characteristics than the parent material. This demonstrates that adding CaCO3 to the AZ31 Mg Alloy enhances its tensile characteristics, with the samples reinforced with 50 % CaCo3 showing the highest tensile rate of 302.43 MPa.

**Table 3:** Tensile test properties.

Sample	Yield Strength (MPa)	Tensile Strength (MPa)	Elongation (%)
A	84.51	302.43	72.06
B	66.24	284.72	76.74
C	200	260.4	15

### 3.3 Hardness test results

To determine the hardness property of the fabricated samples, a load of 100 kgf was applied for a dwell time of 15 s each. Three indentations were made on each sample. The diameter of the indentations as measured through the Brinell microscope were measured and averaged as seen in Table 4.

**Table 4:** Diameter of indentations.

Samples	d1 (μm)	d2 (μm)	d = d2-d1 (μm)	d (mm)
A1	5	6.8	1.8	0.0018
A2	1	2.9	1.9	0.0019
A3	4	5.9	1.9	0.0019
			**Average**	0.00187
B1	1	2.9	1.9	0.0019
B2	2	3.9	1.9	0.0019
B3	4	6	2	0.002
			**Average**	0.00193
C1	2	3.5	1.5	0.0015
C2	1	2.5	1.5	0.0015
C3	4	5.4	1.4	0.0014
			**Average**	0.00147

**Figure 8:**
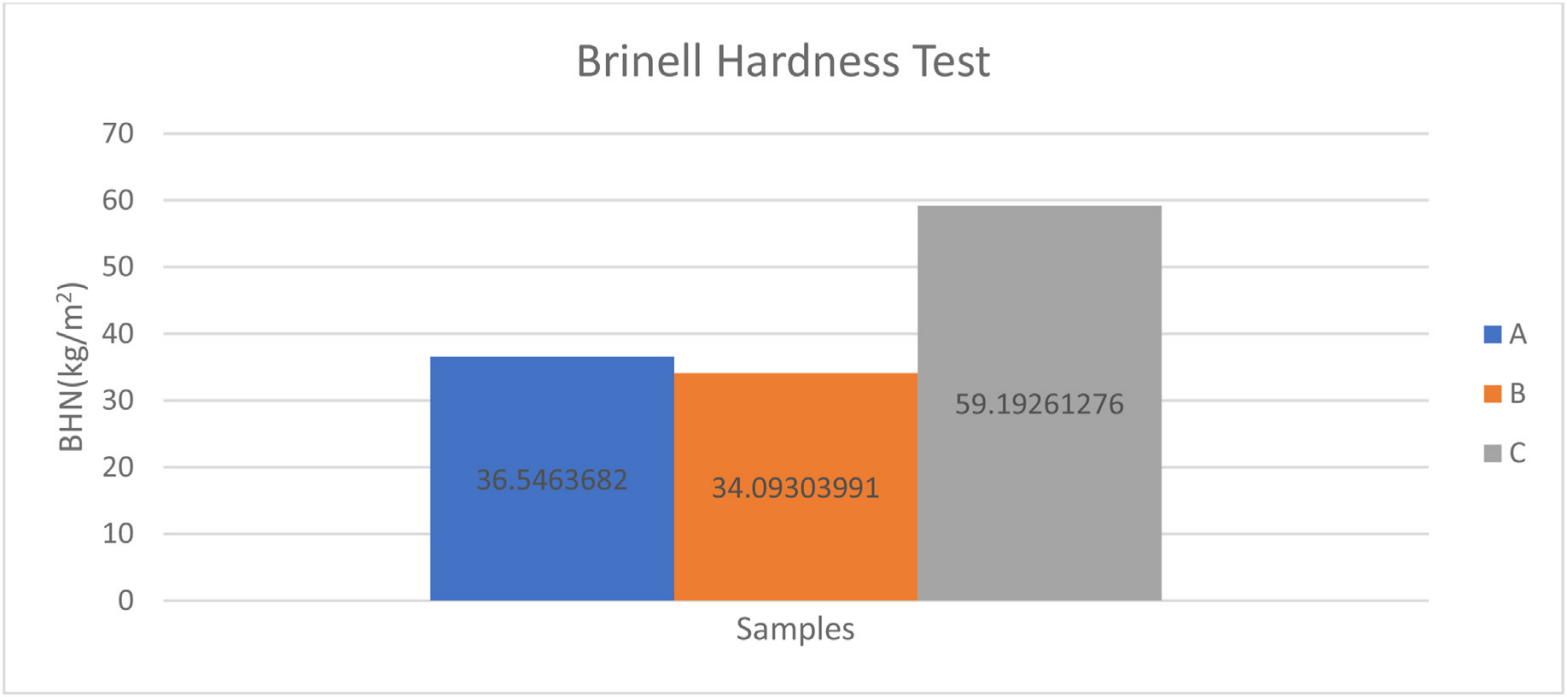
Brinell hardness test values of samples.

The Brinell Hardness Number (BHN) was calculated by using Eq. (1) and the result presented in Table 5. Observations from the chart in Figure 8 indicates that sample C which was not reinforced by CaCO_3_ has the highest hardness property of 59192612.76 kg/mm^2^ when compared to samples A and B. A reduction in hardness values of the reinforced samples could be attributed to the presence of the reinforcement in the composites therefore improving its ductility and reducing hardness [19].

**Table 5:** Hardness test results.

Samples	Load (kgf)	Average Indentation Diameter (mm)	Ball Diameter (mm)	Curved Area (mm^2^)	BHN (kg/mm^2^)
A	100	0.001867	1.57	5.4725E-06	36546368.2
B	100	0.001933	1.57	5.8663E-06	34093039.91
C	100	0.001467	1.57	3.3788E-06	59192612.76

### 3.4 Impact test analysis

**Figure 9:**
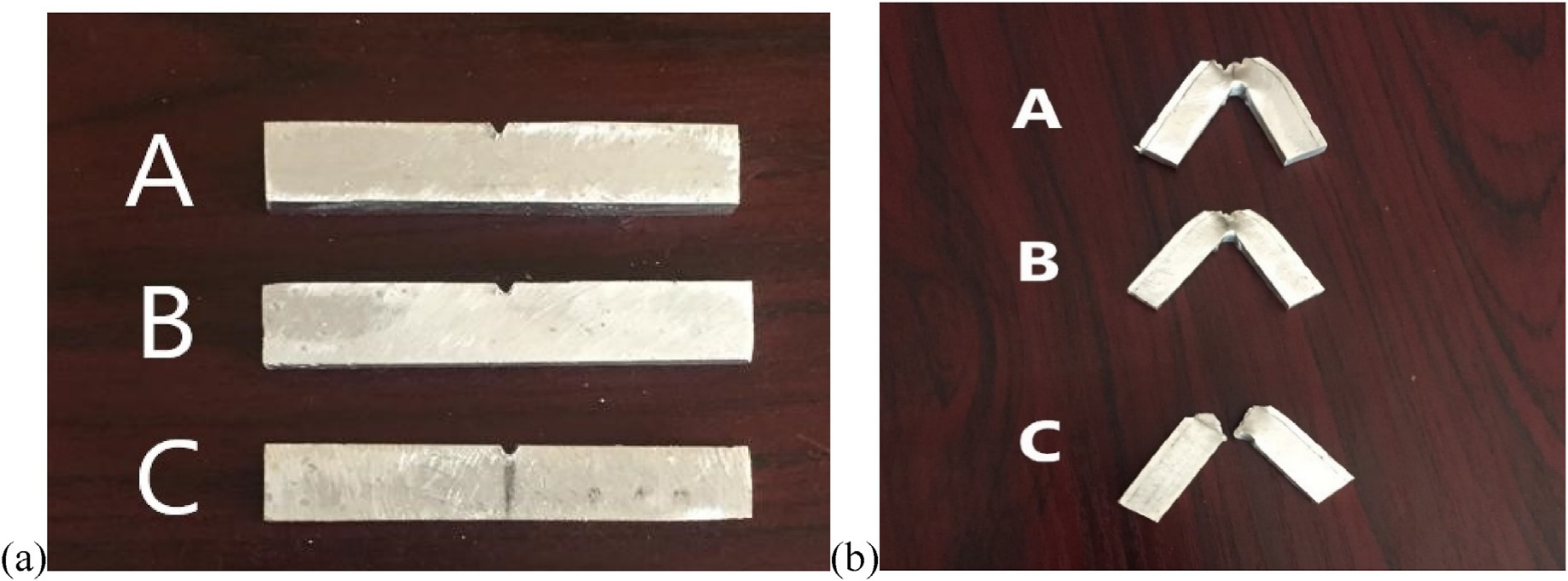
Impact samples (a) before fracture (b) after fracture.

After sample preparations, the samples were fractured by the Charpy Impact Tester. Figures 9a and 9b shows the samples before and after fracture. The types of fracture witnessed in samples A and B are indicators of ductility of the materials.

Table 6 displays the results of the impact test carried out on samples A, B, and C via the Charpy Impact Tester. Sample B (25% reinforcement) was observed to have the highest impact strength 0.1248 **J/mm**^**2**^ when compared to other specimens.

**Table 6:** Impact test result.

S/N	Sample	Energy Absorbed (Joules)	Surface Area of Specimen (mm^2^)	Impact Strength (J/mm^2^)
1	A	135	1490	0.0906
2	B	186	1490	0.1248
3	C	136	1490	0.0913
		**Average Impact Strength**	0.1022 J/mm^2^	

### 3.5 Corrosion test analysis

**Figure 10:**
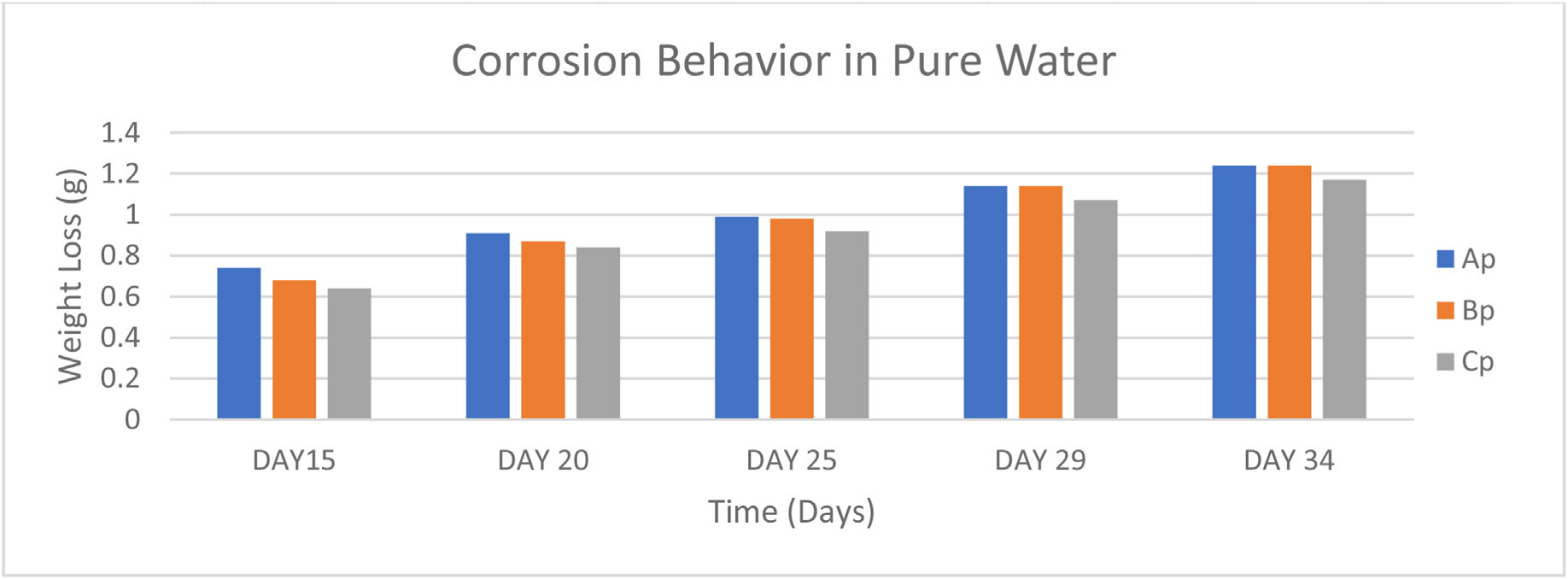
Corrosion behavior in pure water.

For the corrosion test, Fifteen (15) samples in total were obtained after the preparation process, five each of the compositions A, B, and C. The masses of the samples were measured for weight loss over a period of 48 days with intervals of 5 days each. Data from Table 7 shows that the sample B immersed in pure water experienced the greatest weight loss as compared to the other samples. Sample A which was the best performing sample in pure water with the least corrosion rate of 0.02458g/days started out as the fastest corroding specimen in pure water as seen from the chart in Figure 10. However, it was surpassed by Sample B with a corrosion rate of 0.06396g/days and Sample C corroded at a rate of 0.02833g/days.

**Table 7:** Corrosion behavior of samples immersed in pure water.

SAMPLE	DAY 6	DAY 11	DAY 15	DAY 20	DAY 25	DAY 29	DAY 34	DAY 39	DAY 43	DAY 48
Ap	0.47	0.6	0.74	0.91	0.99	1.14	1.24	1.34	1.54	1.65
Bp	0.4	0.54	0.68	0.87	0.98	1.14	1.24	1.31	2.09	3.47
Cp	0.34	0.48	0.64	0.84	0.92	1.07	1.17	1.26	1.44	1.7

**Figure 11:**
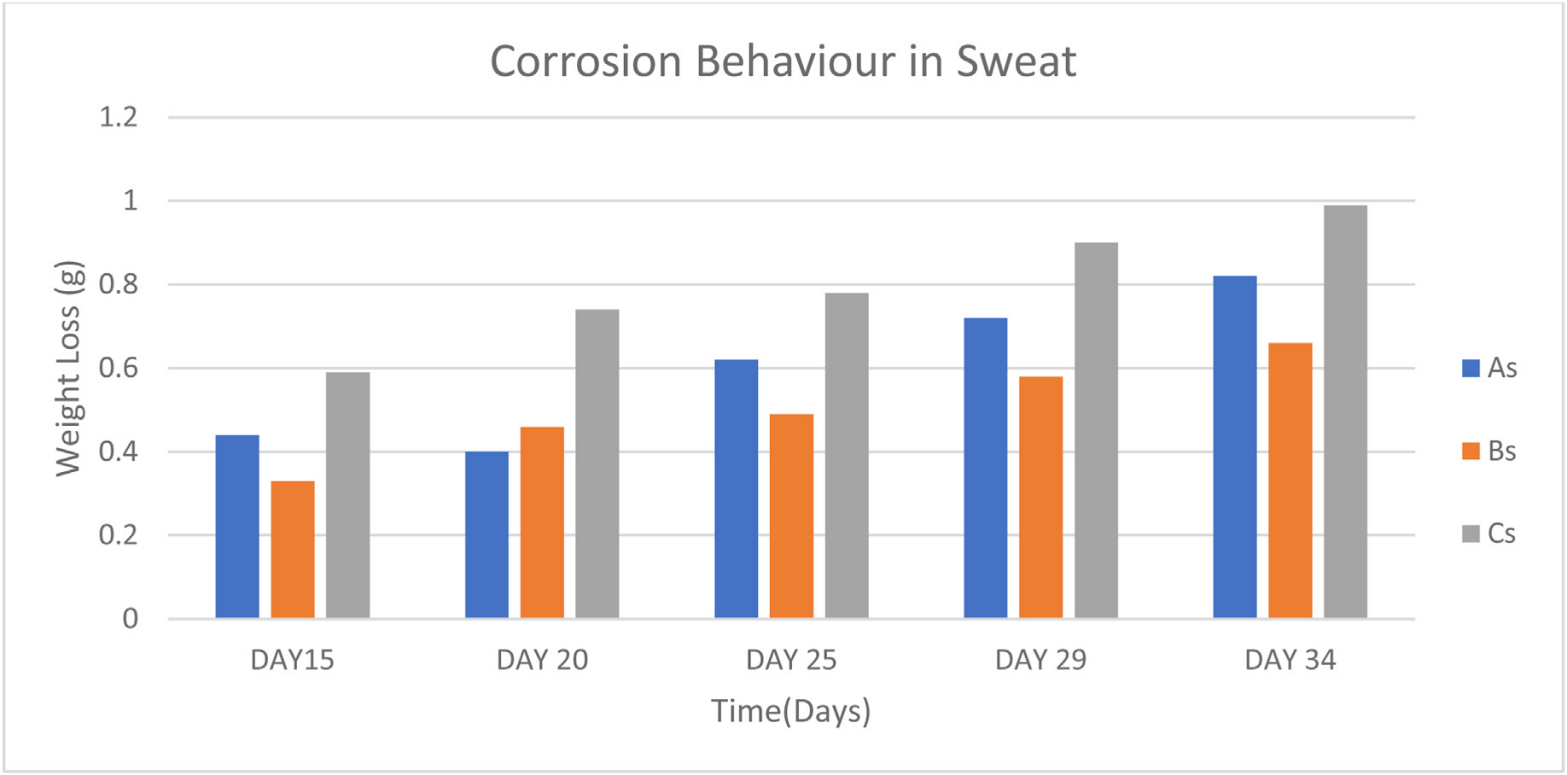
Corrosion behavior in sweat.

The samples immersed in sweat experienced relatively low amounts of weight loss as seen in Table 8 The slowest corrosion rate was observed from Sample B immersed in sweat with a value of 0.01708g/days. Furthermore, Samples A and C surpassed Sample B with corrosion rates of 0.02g/days and 0.01979g/days respectively, as seen from the chart in Figure 11.

**Table 8:** Corrosion behavior of samples immersed in sweat.

SAMPLE	DAY 6	DAY 11	DAY 15	DAY 20	DAY 25	DAY 29	DAY 34	DAY 38	DAY 43	DAY 48
As	0.2	0.31	0.44	0.4	0.62	0.72	0.82	0.93	1.05	1.16
Bs	0.12	0.22	0.33	0.46	0.49	0.58	0.66	0.73	0.8	0.94
Cs	0.28	0.44	0.59	0.74	0.78	0.9	0.99	1.05	1.14	1.23

**Figure 12:**
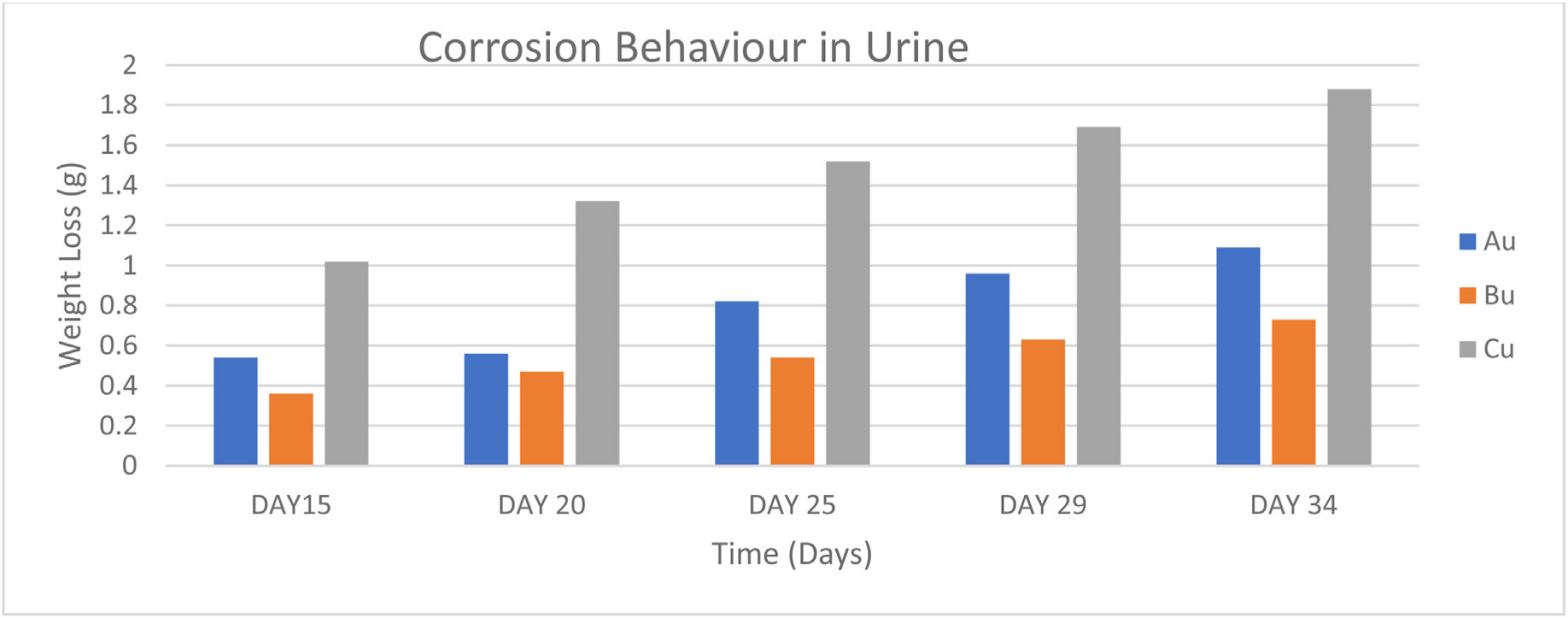
Corrosion behavior in urine.

For the immersion in Urine, Samples A, B and C corroded at rates (0.2208g/days, 0.01729g/days, and 0.04042g/days) respectively according to Table 9. Sample C experienced a significant weight loss in urine as seen in Figure 12, possibly due to undetermined reactions between the material and the medium which led to this rapid corrosion.

**Table 9:** Corrosion behavior of samples immersed in urine.

SAMPLE	DAY 6	DAY 11	DAY 15	DAY 20	DAY 25	DAY 29	DAY 34	DAY 38	DAY 43	DAY 48
Au	0.19	0.34	0.54	0.56	0.82	0.96	1.09	1.17	1.22	1.25
Bu	0.13	0.23	0.36	0.47	0.54	0.63	0.73	0.79	0.85	0.96
Cu	0.07	0.77	1.02	1.32	1.52	1.69	1.88	2.03	2.16	2.01

**Figure 13:**
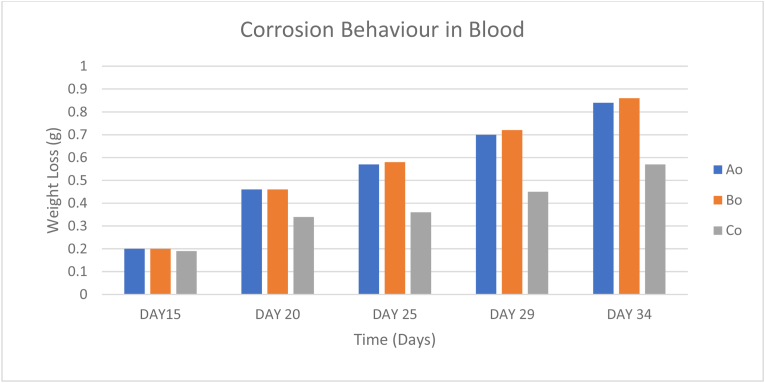
Corrosion behavior in blood.

Similar to the natural water, significant weight losses were observed in blood. Samples A, B, and C experienced corrosion rates of 0.03g/days, 0.02576g/days, and 0.02273g/days respectively. As observed from Table 10 and Figure 13, all three samples corroded at almost equal rates during the first two weeks of testing and then a sudden increase in corrosion rates was experienced from samples A and B.

**Table 10:** Corrosion behavior of samples immersed in blood.

SAMPLE	DAY 6	DAY 11	DAY 15	DAY 20	DAY 25	DAY 29	DAY 34	DAY 38	DAY 43	DAY 48
Ao	-	-	0.2	0.46	0.57	0.7	0.84	0.95	1.05	1.19
Bo	-	-	0.2	0.46	0.58	0.72	0.86	0.96	1.04	1.05
Co	-	-	0.19	0.34	0.36	0.45	0.57	0.69	0.81	0.94

**Figure 14:**
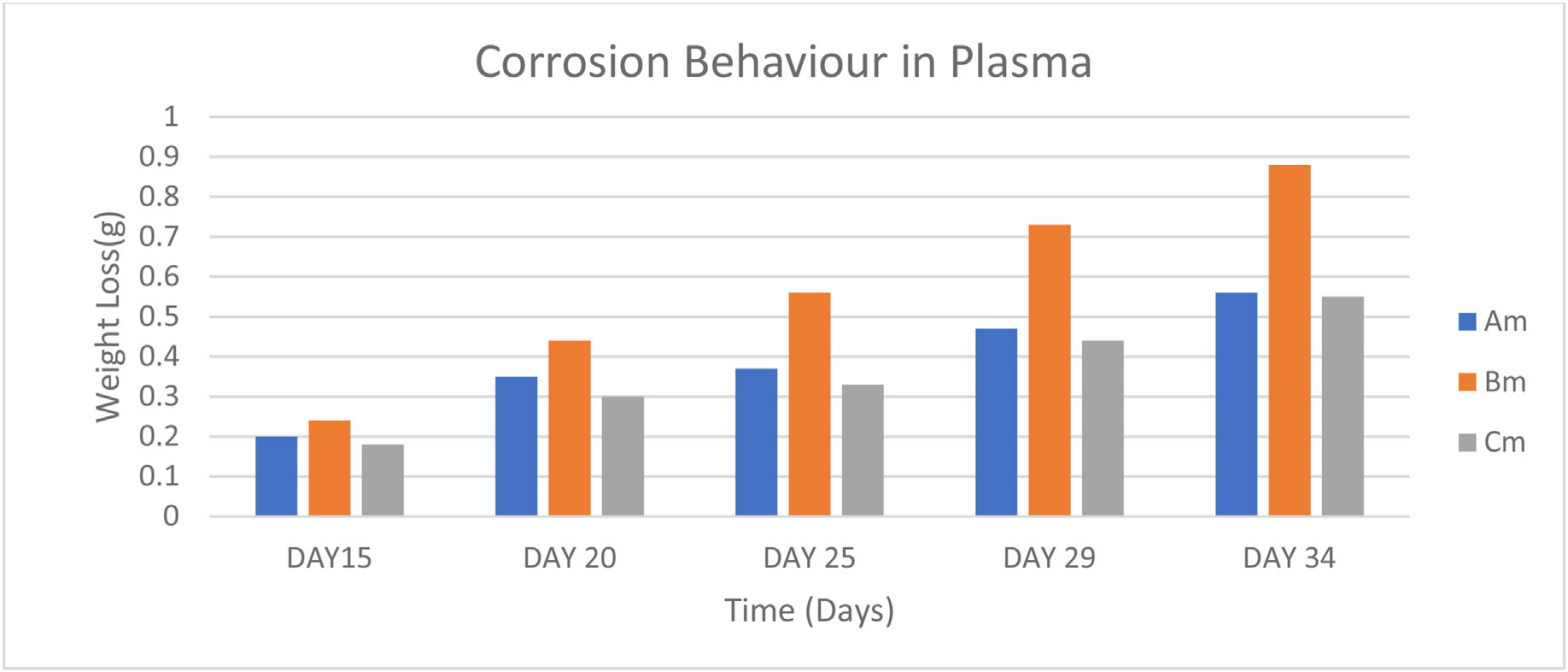
Corrosion behavior in plasma.

Plasma was used as the simulated body fluid, which is a combination of blood and water often found around bones such as femur and humerus bones. The result shows that low corrosion rates (0.01788g/days, 0.03121g/days, and 0.01727g/days) of Samples A, B, and C respectively were experienced in plasma as shown in Table 11. Figure 14 shows that Sample B actively degraded in the medium compared to the other samples.

**Table 11:** Corrosion behavior of samples immersed in plasma.

SAMPLE	DAY 6	DAY 11	DAY 15	DAY 20	DAY 25	DAY 29	DAY 34	DAY 38	DAY 43	DAY 48
Am	-	-	0.2	0.35	0.37	0.47	0.56	0.62	0.71	0.79
Bm	-	-	0.24	0.44	0.56	0.73	0.88	1.01	1.14	1.27
Cm	-	-	0.18	0.3	0.33	0.44	0.55	0.62	0.69	0.75

Observations made from the corrosion test shows that Sample A was averagely the slowest degrading sample across all corrosive media. Consequently, Sample B was averagely the worst performing sample amongst all three samples. Specifically, according to the result of the immersion in various corrosive media, the greatest corrosion was observed in Sample B immersed in natural water with a corrosion rate of 0.06395g/days while the least corrosion was observed in Sample B immersed in sweat solution with a rate of 0.01708g/days (0.0001752 g/year). Therefore, the reinforcements improved the corrosion resistance of the AZ31B Mg alloy. The sample A showing lowest corrosion rate can be seen as a good implant with long degradability within human body.

## 4 Conclusion

The focus of this study was to investigate the properties of AZ31B magnesium alloy reinforced with CaCO_3_ powder as an orthopedic implant. The AZ31 Mg alloy was successfully strengthened with CaCO_3_ powder via stir casting technique in this study. The need for a second surgery after a broken bone has been aligned with the help of an implanted material is the driving factor behind this study.

The newly fabricated AZ31B Mg/CaCO3 composites were analyzed, and the following conclusions were:

• Stir casting is an efficient method for developing metal matrix composites.
• AZ31B Mg/CaCO_3_ has a good chance of being employed in orthopedic applications where corrosion resistance is critical.
• The tensile characteristics and corrosion resistance of samples A (50% reinforced) and B (25% reinforced) were both enhanced.
• The reinforcing particles being Calcium Carbonate, has good biocompatibility with human bones hence help with the healing process.

## Declarations

### Author contribution statement

Adedotun Adetunla: Conceived and designed the experiments; Analyzed and interpreted the data; Wrote the paper.

Anthony Fide-Akwuobi: Performed the experiments; Analyzed and interpreted the data.

Henry Benjamin: Conceived and designed the experiments.

Adebayo Adeyinka, Adenike Kolawole: Contributed reagents, materials, analysis tools or data.

### Funding statement

This research did not receive any specific grant from funding agencies in the public, commercial, or not-for-profit sectors.

### Data availability statement

Data included in article/supplementary material/referenced in article.

### Declaration of interests statement

The authors declare no conflict of interest.

### Additional information

No additional information is available for this paper.
